# Health of university students under job and financial insecurity during COVID‐19 pandemic

**DOI:** 10.1002/1348-9585.12223

**Published:** 2021-03-31

**Authors:** Hiroshi Yatsuya, Tatsuya Ishitake

**Affiliations:** ^1^ Department of Public Health and Health Systems Nagoya University Graduate School of Medicine Nagoya Aichi Japan; ^2^ Department of Environmental Medicine Kurume University School of Medicine Kurume Fukuoka Japan

Enrolling rate to higher education including university, junior college, and vocational school in Japan is continuously increasing and was 83.5% in 2020.^1^ And the number of university students as of May 2020, approximately 2.6 million, is also the largest ever. According to the annual survey conducted by Japan National Federation of University Cooperative Associations from October to November 2020, the proportion of university students working part‐time declined sharply to 72.4% in the first half fiscal year of 2020 from 83.9% in 2019.^2^ The same survey reported that the earning from part‐time job accounts for approximately 60% of the income for students living with the family and for one‐fifth to one‐fourth for students living away from the family. The decline was observed not only in the number of students working part‐time but in the amount of earning although 61.9% of the university students in the survey reported that their life was better‐off in spite of the fact that one‐fourth of the survey respondents foresaw financial hardship in near future. It is still difficult to summarize the multifaceted influence of the global COVID‐19 pandemic, but university students would be one of the most affected groups in the population. From occupational health point of view, these situations encountered by university students may represent that of nonregular workers in general, on which the pandemic has brought differential negative impact. The proportion of nonregular employment in 2020 decreased for the first time in the last decade, indicating their vulnerability of the job security.^3^, ^4^

In the present issue of the Journal of Occupational Health, Tsurugano et al. reported that nearly half of working students lost their jobs as of late May 2020 according to the online nonanonymous survey of living conditions conducted at a national science and engineering university in Tokyo.^5^ The survey included 2530 responses, which accounts for approximately 50% of the students consisting of 70 % undergraduates and 90% men. They also identified 37.4% of students were financially insecure. The report provided context analyses of free text entries, and extracted the following five broad response categories: income, job search, financial support, continuing studies, and health. They are literally very impressive and indicates urgent needs to identify those vulnerable students as well as the countermeasures. Indeed, they found that students with economic insecurity were more likely to have poor self‐rated health compared to those who were financially stable.

A similar survey conducted in Akita prefecture found 23% of male and 20% of female university students in the financial insecurity, which was associated with 2.79 times higher risk of suicide‐related ideation.^6^ This finding is worrisome as monthly suicide rates reportedly increased by 16% after the first state of emergency from July to October 2020 in Japan, with a larger increase among females and children and adolescents.^7^ Furthermore, a meta‐analysis of 27 studies published by July 27, 2020, that included 90,879 college students reported that anxiety and depression are quite prevalent among college students (39.4% and 31.2%, respectively).^8^ The study also found high pooled prevalence of stress (26.0%), posttraumatic stress disorder (29.8%), and impaired sleep quality (50.5%).

As Tsurugano et al. concluded, the sudden economic insecurity brought about by the global COVID‐19 chaos would have long‐lasting consequences and regrettable loss of young generation unless sufficient public support is provided as it is likely to be having a devastating effect on their studies and health.
